# Cancer worry and its impact on self-reported depressive symptoms among adult males and females in the US: a nationwide sample study

**DOI:** 10.1186/s12888-023-05405-4

**Published:** 2024-01-08

**Authors:** Lohuwa Mamudu, Jinyi Li, Archana J. McEligot, Michele Wood, Pimbucha Rusmevichientong, Erasmus Tetteh-Bator, Abdul-Nasah Soale, James D. Fortenberry, Faustine Williams

**Affiliations:** 1 Department of Public Health, California State University, 800 N. State College Boulevard, Fullerton, Fullerton, CA 92831 USA; 2 Department of Public Health, University of California Irvine, Irvine, CA 92967 USA; 3 Department of Mathematics and Statistics, University of South Florida, 4202 E. Fowler Ave, Tampa, FL 33620 USA; 4 Department of Mathematics and Statistics, Applied Mathematics and Statistics, Case Western Reserve University, Yost Hall, 2049 Martin Luther King Jr. Drive, 44106-7058 Cleaveland, OH USA; 5 Division of Adolescent Medicine, Indiana University School of Medicine, 410 W 10th St., Room 1001, Indianapolis, IN 46202 USA; 6 Division of Intramural Research, National Institute on Minority Health and Health Disparities, National Institutes of Health, 11545 Rockville Pike, T-10 C12, Rockville, MD 20852 USA

**Keywords:** Depressive symptoms, Cancer worry, Males & females, Chronic Disease, Psychiatric Disorder

## Abstract

**Objective:**

With cancer the second deadliest disease in the world, worry about cancer can have mental health or psychiatric implications. This study examines the prevalence, differences, and influence of cancer worry (CW), its interaction effect with age, and other confounders on self-reported depressive symptoms (SRDS) among adult males and females in the US.

**Methods:**

We utilized a nationally representative sample data of 2,950 individuals (males = 1,276; females = 1,674) from Cycle 4 of the Health Information National Trends Survey 5 (HINTS 5) 2020. Using frequencies, bivariate chi-square test, and multivariate logistic regression, we examined the prevalence, difference, and association of CW with SRDS, adjusting for confounders.

**Results:**

The prevalence rate of SRDS was found to be 32% among females and 23.5% among males. Among individuals with CW, females had a higher prevalence of SRDS compared to males (40.5% vs. 35.1%). However, there was a significant difference in the likelihood of experiencing SRDS between males and females with CW, with males having 84% increased risk compared to females. Across all age groups, the multivariate analysis of the relationship between CW and SRDS revealed that both males and females showed a significantly decreased likelihood of SRDS compared to those aged 18–34 years. However, males aged 35 years or older exhibited an even more pronounced decrease in likelihood compared to females in the same age group. Nonetheless, when examining the interaction of age and CW, we observed a significantly increased likelihood of SRDS across all age groups. Males, in particular, had a higher increased likelihood of SRDS compared to females across all ages, except for those aged 75 years and older.

**Conclusion:**

The findings of this study highlight the significant influence of CW on individuals’ SRDS and the modifying effect of age, particularly among males. These results are important for a better understanding of the risk of CW on mental health, which can be a preventive strategy or control mechanism.

## Introduction

Cancer is one of the most stressful, anxiety-provoking, and fearsome diseases in the world [1, 2]. The possibility of cancer diagnosis may have mental health consequences. Cancer patients or people historically at risk of getting cancer have been shown to be at a higher risk of mental health issues such as depression and anxiety [3–5]. For instance, the worry about cancer may result in cancer anxiety, fear of cancer, cancer-related distress, and cancer-specific distress [6]. Clemow et al. (2000) found that breast cancer worry (CW) predicted stronger plans to obtain a mammogram, but fear of testing positive was negatively associated with mammography intentions [7]. While CW can lead to positive outcomes such as increased engagement in self-protective behaviors like cancer screening [8], the negative consequences of cancer worry tend to outweigh the positives. Research has shown that CW can result in distress and avoidance of screening, which can be problematic [9, 10]. Kash et al. (1992) found in a discriminant function analysis that increased cancer anxiety was associated with decreased clinical examinations [9]. Furthermore, cancer significantly affects the quality of life of patients and exacerbates existential concerns, including anxiety and depressive symptoms [11]. The needs and concerns about cancer extend beyond the immediate treatment of the disease, but also have emotional, interpersonal, and social implications for patients as well as their family members [12].

Depressive symptoms may precede serious medical illnesses such as cancer due to the worry, anxiety, nervousness, and fear of being diagnosed with the disease. The worry about cancer may also differ by gender. McQueen et al. (2008) found that women reported more worry about cancer than men [13]. Additionally, age has been identified as a factor that can impact the risk of cancer, which in turn may influence the level of cancer-related worry. Mary et al. (2014) reported age to be associated with chronic conditions, exposures, and risk behaviors that are causally associated with cancer [14]. These risk factors for cancer have also been shown to be associated with depression. For instance, studies have shown some differences and the likelihood of depression between male and female patients with cancer [15, 16]. Angst et al. (2002) found that gender differences in major depression persisted across all age groups [17]. Also, men reported fewer symptoms and had increased coping mechanisms than women [17]. Therefore, it is imperative to distinctively assess the influence of CW on depressive symptoms to advocate for more effective policy intervention. Further, it is unclear how age may modify the relationship between CW and the likelihood of depressive symptoms. No study has exclusively examined the influence of age as effect modifier on the relationship between CW and depressive symptoms within and between males and females among US adults. Consequently, it is important to understand how the interaction of CW with various age groups influences the likelihood of experiencing major depressive symptoms.

In this study, we assessed and compared the association between CW and self-reported depressive symptoms (SRDS) among adult males and females in the US. Other specific aims are: (1) to examine the prevalence of SRDS with CW and other confounders (e.g., sociodemographic and socioeconomic characteristics and smoking behavior); (2) to investigate the difference in SRDS among the levels of frequent worried about cancer and other confounders; (3) to assess the likelihood of SRDS association with CW and other confounders; and (4) to investigate the likelihood of SRDS with CW interaction across all age groups and genders. We hypothesize that CW will be associated with SRDS with some underlying disparities, and the interaction of CW and age may modify the influence of SRDS. Addressing these conditions could potentially mitigate risk factors and the adverse outcomes associated with depressive symptoms, as well as other mental health disorders such as sadness, helplessness, hopelessness, suicidal ideation, and chronic diseases such hypertension.

## Materials and methods

### Study design and date source

We obtained secondary data from the Health Information National Trends Survey (HINTS). HINTS is a yearly cross-sectional survey of the non-institutionalized nationally representative adult population in the United States [18–20]. We used the most recent HINTS data, HINTS 5 Cycle 4 data, collected from February to June 2020. Access to HINTS data does not require Institutional Review Board approval. The data have been de-identified and made accessible for public use through the National Cancer Institute (NCI) website, https://hints.cancer.gov. HINTS uses two-stages sampling survey design. In stage (1), an equal probability sample of respondents is selected within each explicit sampling stratum, and in stage (2), one adult was chosen within each sampled household. A questionnaire was considered complete if respondents answered ≥ 80% of the questions and partially complete if 50–70% were answered [18]. The HINTS 5 Cycle 4 data included a total of 3,865 respondents, including missing data, nonresponse, and response error. The data samples are weighted, and oversampling was used to increase the sample from minority populations. We conducted the Cronbach’s α and found it to be 0.85, indicating that our data is reliable. All participants with at least one missing data of variable of interest were excluded from our final analysis. Our final sample included 2,950 respondents with complete information on CW and SRDS measured using the Patient Health Questionnaire (PHQ-4). An extensive description of HINTS data study and methodologies has been published elsewhere [18, 20]. The data included self-reported adults’ personal information, health-related information, and health behaviors. For the present study, the variables accessed included depressive symptoms, sociodemographic and socioeconomic characteristics, health and behavioral risk factors, and CW.

### Measures

#### Outcome/Dependent variable

Self-reported depressive symptoms is the primary outcome variable. This was derived from PHQ-4 with a total score ranging from 0 to 12. To assess different levels of depressive symptoms, the PHQ-4 score is often categorized as none/normal (0–2), mild (3–5), moderate (6–8), and severe (9–12) [21–23]. To achieve the objective of this study, the PHQ-4 was recategorized into two as “none/normal depressive symptoms” for individuals with PHQ-4 score of 0–2; and “moderate/severe depressive symptoms” for individuals with PHQ-4 of 3–12 [24, 25].

#### Independent variables/Exposures

The exposure or main independent variable is CW. HINTS asked individuals “How worried are you about getting cancer?” with the response options not at all; slightly; somewhat; moderate; or extremely worried. We dichotomized this variable as “none/normal worry” [Ref.] for individuals who responded not at all/slightly/somewhat worry; and “moderate/serious” for those who responded moderate/extremely worried. A similar classification was adopted by Andersen et al. (2003) in a study on breast cancer worry and mammography use [26].

#### Confounders

Other potential independent variables or confounders include sociodemographic and socioeconomic characteristics and smoking behavior. The sociodemographic characteristics included sex at birth (male or female), age (18–34 [Ref.]; 35–49 50–64 65–74, 75]), race/ethnicity (non-Hispanic White [Ref.]; non-Hispanic Black/African American; Hispanic; non-Hispanic Asian; non-Hispanic Others), marital status (single/never married [Ref.]; married/living as married or living with a romantic partner; divorced/separated; widowed), and sexual orientation (heterosexual/straight [Ref.]; homosexual/gay/lesbian; bisexual). Socioeconomic characteristics include education level (Less than High School [Ref.]; High school graduate; ​​some college; college graduate/More), employed status (yes or no [Ref.]), household income (less than $20,000 [Ref.]; $20,000 to < $35,000; $35,000 to < $50,000; $50,000 to < $75,000; $75,000+), and health insurance status (yes or no [Ref.]). The lifestyle risk variable is smoking status (never [Ref.]; current; former).

### Statistical analysis

Individuals in the data with nonresponses or missing data were excluded from our analysis using the listwise deletion method, resulting in complete data of 2,950 individuals, which is 76% of the original sample. We then conducted the Cronbach’s α to assess the reliability of our data. Firstly, we estimated the prevalence rate, the absolute difference, and the relative/odds ratio of SRDS among males and females who worry about cancer. Descriptive statistics, including cross-tabulation frequencies are presented to estimate the prevalence rate of SRDS among subgroup samples stratified by males and females. We conducted a bivariate analysis using the Chi-square (${\chi }^{2}$) test to assess the statistical difference in SRDS among CW and confounders. Results of the Chi-square test are reported based on the statistical significance of *p*-value < 0.05 level of significance. Finally, comparative multivariate analysis using logistic regression models were performed to examine the extent of association of CW and confounders with SRDS. Model I assessed the association among males, Model II assessed the association among females, and Model III assessed the association of interaction between age and CW with SRDS by a stratified sample of males and females, respectively, adjusting for other confounders. The stepwise model selection procedure was adopted to select the best models with minimum sampling and predictive error. Results from the logistic regression models are reported using adjusted odds ratios (AORs), 95% confidence intervals (CIs) of the AORs, and a statistical significance level of *p*-value < α = 0.05, 0.01, 0.001. Finally, we displayed cluster bar plots to show the interacting effect of CW and age on SRDS. All statistical analyses in this study were performed using IBM SPSS Statistics Software Version 28.

## Results

### Prevalence of self-reported depressive symptoms among males and females and cancer worry

Table 1 presents the prevalence rate, absolute difference, and relative/odds ratio of depressive symptoms among males and females with CW in the US. Females who were moderately/seriously worried about cancer consistently had a higher prevalence rate at all levels of depressive symptoms than males, i.e., mild (males = 20.7%, females = 20.9%), moderate (males = 7.9%, females = 11.2%), and severe (males = 6.5%, females = 8.4%). Also, the prevalence of moderate depressive symptoms was 1.42 times higher among females with CW than males. Similarly, the prevalence of severe depressive symptoms was 1.29 times higher among females with CW than their male counterparts.

**Table 1:** Prevalence of depressive symptoms among US adult male and female with cancer worry

	Male	Female		
Depressive Symptoms	Cancer Worry	Cancer Worry	Absolute	Relative
	[Moderate/Serious %] (95% CI)	[Moderate/Serious %] (95% CI)	Difference	Risk Ratio
None	64.9 (55.2–73.7)	59.5 (49.7–68.7)	5.4	0.92
Mild	20.7 (13.7–29.4)	20.9 (13.8–29.6)	0.2	1.01
Moderate	7.9 (3.8–14.4)	11.2 (6.1–18.5)	3.3	1.42
Severe	6.5 (2.9–12.6)	8.4 (4.1–15.0)	1.9	1.29

HINTS 5 Cycles 4 data was collected from February through June 2020. Unweighted sample *N* = 2,950 and weighted sample *N* = 44,546,288

### Examination of prevalence and statistical difference in self-reported depressive symptoms

Table 2 examines the prevalence and statistical differences in SRDS. The prevalence rate of SRDS for males and females was 23.5% and 32.0%, respectively. There were several similarities in terms of the highest prevalence rate of depressive symptoms within the subgroup of male and female adults in the U.S. Within the subpopulation sample, we found the highest prevalence rates of SRDS was among individuals who experience CW (males: 35.1%,females: 40.5%); those aged 18–34 years (males = 34.9%, females = 41.5%) among age groups; non-Hispanic other (males = 37.2%, females = 47.7%) among race/ethnicity; bisexual (males = 50%, females = 58.2) among sexual orientation; less than high school education (males = 30.7%, females = 39.4%); employed (males = 25.9%, females = 32.2%); earning less than $20,000 annual household income (males = 40.3%, females = 44.8%); with no health insurance (males = 25.8%, females = 36.8%); and those who currently smoke (males = 33.1%, females = 47.5%).

**Table 2:** Descriptive characteristics, prevalence, and difference in self-reported depressive symptoms by independent variables (*N* = 2,950)

	Male			Female		
	Total	Depressive	*P*-Value	Total	Depressive	*P*-Value
	*N* (%)	Symptoms		*N* (%)	Symptoms	
		***n*** (%)			***n*** (%)	
**Independent Variables**	1,276 (43.3)	300 (23.5)		1,674 (56.7)	536 (32.0)	
**Cancer Worry**			< 0.001***			< 0.001***
None/Normal	923 (72.3)	176 (19.1)		1163 (69.5)	329 (28.3)	
Moderate/Serious	353 (27.7)	124 (35.1)		511 (30.5)	207 (40.5)	
**Confounders**						
**Sociodemographics**						
**Age**			< 0.001***			< 0.001***
18–34	166 (13.0)	58 (34.9)		270 (16.1)	112 (41.5)	
35–49	232 (18.2)	56 (24.1)		381 (22.8)	135 (35.4)	
50–64	417 (32.7)	105 (25.2)		429 (29.6)	159 (32.1)	
65–74	321 (25.2)	61 (19.0)		348 (20.8)	76 (21.8)	
75+	140 (11.0)	20 (14.3)		179 (10.7)	54 (30.2)	
**Race/Ethnicity**			0.269			0.002**
Non-Hispanic White	807 (63.2)	184 (22.8)		1039 (62.1)	331 (31.9)	
Non-Hispanic Asian	77 (6.0)	17 (22.1)		62 (3.7)	12 (19.4)	
Non-Hispanic Black/African American	136 (10.7)	30 (22.1)		232 (13.9)	61 (26.3)	
Hispanic/Latino	213 (16.7)	53 (24.9)		284 (17.0)	105 (37.0)	
Non-Hispanic Other/Multi-Racial	43 (3.4)	16 (37.2)		57 (3.4)	27 (47.4)	
**Marital Status**			< 0.001***			0.047*
Single/Never Married	219 (17.2)	68 (31.1)		298 (17.8)	107 (35.9)	
Married/living as Married	781 (62.0)	156 (19.7)		834 (49.8)	247 (29.6)	
Divorced/Separated	197 (15.4)	21 (30.4)		324 (20.4)	124 (36.3)	
Widowed	69 (5.4)	55 (27.9)		200 (11.9)	58 (29.0)	
**Sexual Orientation**			< 0.001***			< 0.001***
Heterosexual/Straight	1211 (94.9)	271 (22.4)		1592 (95.1)	490 (30.8)	
Bisexual	20 (1.6)	10 (50.0)		55 (3.3)	32 (58.2)	
Homosexual/Gay/Lesbian	45 (3.5)	19 (42.2)		27 (1.6)	14 (51.9)	
**Socioeconomics**						
**Level of Education**			0.013*			0.086
Less than High School	75 (5.9)	23 (30.7)		109 (6.5)	43 (39.4)	
High School Graduate	199 (5.6)	58 (29.1)		283 (16.9)	100 (35.3)	
Some College	391 (30.6)	98 (25.1)		482 (28.8)	157 (32.6)	
College Graduate/more	611 (47.9)	121 (19.8)		800 (47.8)	236 (29.5)	
**Employment status**			0.070			0.346
Yes	563 (44.1)	146 (25.9)		890 (46.8)	260 (32.2)	
No	713 (55.9)	154 (21.6)		784 (46.8)	276 (31.0)	
**Household Income**			< 0.001***			< 0.001***
<$20,000	159 (12.5)	64 (40.3)		286 (17.1)	128 (44.8)	
$20,000 to <$35,000	134 (10.5)	33 (24.6)		236 (14.1)	85 (36.0)	
$35,000 to <$50,000	1 59 (12.5)	36 (22.6)		229 (13.7)	69 (30.1)	
$50,000 to <$75,000	241 (18.9)	59 (24.5)		281 (16.8)	94 (33.5)	
≥$75,000	583 (45.7)	108 (18.5)		642 (38.4)	160 (24.9)	
**Health Insurance**			0.662			0.328
Yes	1214 (95.1)	284 (23.4)		1587 (94.8)	504 (31.8)	
No	62 (4.91)	16 (25.8)		87 (5.2)	128 (36.8)	
**Health Behavior**						
**Smoke status**			0.004**			< 0.001***
Never	736 (57.7)	154 (20.9)		1132 (67.6)	326 (28.8)	
Current	163 (12.8)	54 (33.1)		177 (10.6)	8 (47.5)	
Former	377 (29.5)	92 (24.4)		365 (21.8)	126 (34.5)	

HINTS 5 Cycles 4 data was collected from February through June 2020. Frequencies and prevalence are estimated from the weighted samples of 44,546,288 U.S. household. Bold values: Statistical significance with *p < 0.05, **p < 0.01, ***p < 0.001

In the marital group, we found that single/never married individuals had the highest prevalence of SRDS among males (31.1%) compared to divorced among females (36.3%). Overall, females exhibited a higher prevalence rate of SRDS compared to their male counterparts across various independent variables and confounders. However, among non-Hispanic Asians, males experienced a higher SRDS prevalence rate (males = 22.1%, females = 19.4%).

The assessment of statistical differences in SRDS showed the presence of significant differences among individuals with CW for both male and female subgroups and among the following confounders: age group, marital status, sexual orientation, household income, and smoking status. In addition, there was a significant difference in race/ethnicity among females and the level of education among males. However, no statistical difference in SRDS was found among males’ race/ethnicity and the level of education among females.

### Assessment of Likelihood of self-reported depressive symptoms association with Independent factors (model I-male and II-female)

Table 3 shows the results of the multivariate logistic regression analysis examining independent factors associated with SRDS. Across all age groups, both males and females showed a significantly decreased likelihood of SRDS compared to those aged 18–34 years. However, males aged 35 years or older exhibited an even higher decreased likelihood (AOR = 0.15–0.51) compared to females in the same age group (AOR = 0.41–0.77). Further, non-Hispanic Black/African American females were 31% (AOR = 0.69; 95% CI = 0.49–0.98) less likely to report SRDS compared to non-Hispanic White females, which was higher than 19% decrease observed among their male counterparts, though this difference was not statistically significant. Homosexual/gay/lesbian individuals of both genders had a higher likelihood of reporting depressive symptoms, with males exhibiting a significant likelihood than females (males: AOR = 2.31, 95% CI = 1.19–4.49 vs. females: AOR = 2.06, 95% CI = 1.14–5.65). Similarly, bisexual males and females had a higher likelihood of reporting SRDS than heterosexual/straight respondents, but females showed a higher odd than males (males: AOR = 3.02, 95% CI = 1.14–7.98 vs. females: AOR = 3.85, 95% CI = 1.22–3.98). Individuals earning a household annual income ≥$20,000 had a lower likelihood of association with SRDS for both males and females, but males were often more likely than females (males: AOR = 0.48–0.52 vs. females: AOR = 0.40–0.75). Further, males who are not employed had a higher likelihood of reporting depressive symptoms than those employed (AOR = 1.56, 95% CI = 1.08–2.25), but females had a lower likelihood (AOR = 0.99, 95% CI = 1.00 = 1.69). Both males and females with CW tend to have a higher association with SRDS (males: AOR = 2.44; 95% CI = 1.82–3.26 vs. female: AOR = 1.60; 95% CI = 1.27–2.02 vs.), but males were more likely than females. Both former and current smokers, regardless of gender had an increased likelihood of being associated with SRDS. However, current female smokers were more likely than their male counterparts (males: AOR = 1.37, 95% CI = 0.90–2.08 vs. females: AOR = 1.65, 95% CI = 1.15–2.35), while former male smokers showed a higher likelihood than their female counterparts (males: AOR = 1.42, 95% CI = 1.02–1.96 vs. females: AOR = 1.37, 95% CI = 1.04–1.79).

**Table 3:** Multivariate logistic regression analysis of independent factors association with self-reported depressive symptoms

	Model I: Male	Model II: Female
Independent Variables/Covariates	AOR (95% CI)	AOR (95% CI)
Cancer Worry		
None/Normal [Ref]	-	-
Moderate/Serious	2.44 (1.82, 3.26)***	1.60 (1.27, 2.02)***
**Confounders**		
**Sociodemographics**		
**Age**		
18–34 [Ref]	-	-
35–49	0.51 (0.32, 0.83)**	0.77 (0.54, 1.10)
50–64	0.41 (0.26, 0.65)***	0.60 (0.42, 0.86)***
65–74	0.24 (0.14, 0.41)***	0.28 (0.18, 0.43)****
75+	0.15 (0.07, 0.30)***	0.41 (0.24, 0.69)***
**Race/Ethnicity**		
Non-Hispanic White [Ref]	-	-
Non-Hispanic Asian	1.02 (0.55,1.87)	0.58 (0.30, 1.13)
Non-Hispanic Black/African American	0.81 (0. 51, 1.31)	0.69 (0.49, 0.98)*
Hispanic/Latino	0.84 (0.57, 1.25)	1.0 4 (0.76, 1.41)
Non-Hispanic Other/Multi-Racial	1.70 (0.85, 3.38)	1.63 (0.92, 2.89)
**Marital Status**		
Single/Never Married [Ref]	-	-
Married/living as Married	1.02 (0.67, 1.54)	1.03 (0.74, 1.42)
Divorced/Separated	1.67 (0.85, 3.29)	1.12 (0.70, 1.78)
Widowed	1.39 (0.85, 2.28)	1.26 (0.87, 1.84)
**Sexual Orientation**		
Heterosexual/Straight [Ref]	-	-
Bisexual	3.02 (1.14, 7.98)*	3.85 (1.22, 3.98)**
Homosexual/Gay/Lesbian	2.31 (1.19, 4.49)*	2.06 (1.14, 5.65)*
**Socioeconomics**		
**Level of Education**		
Less than High School [Ref]	-	-
High School Graduate	1.05 (0.56, 1.95)	0.97 (0.59, 1.59)
Some College	0.89 (0.48, 1.60)	0.86 (0.5 3, 1.39)
College Graduate/more	0.75 (0.40, 1.38)	0.95 (0.58, 1.55)
**Employment status**		
Yes [Ref]	-	-
No	1.56 (1.08, 2.25)*	0.99 (1.00, 1.69)
**Household Income**		
<$20,000 [Ref]	-	-
$20,000 to <$35,000	0.57 (0.32, 0.99)*	0.75 (0.51, 1.10)
$35,000 to <$50,000	0.57 (0.33, 0.98)*	0.58 (0.38, 0.85)**
$50,000 to <$75,000	0.62 (0.38, 1.04)	0.62 (0.42, 0.92)*
≥$75,000	0.48 (0.29, 0.79)**	0.40 (0.27, 0.60)***
**Health Insurance**		
Yes [Ref]	-	-
No	0.72 (0.38, 1.37)	0.73 (0.45, 1.19)
**Health Behavior**		
**Smoke status**		
Never [Ref]	-	-
Current	1.37 (0.90, 2.08)	1.65 (1.15, 2.35)**
Former	1.42 (1.02, 1.96)*	1.37 (1.04, 1.79)*

HINTS 5 Cycles 4 data was collected from February through June 2020. *AOR* Adjusted odds ratio, *95% CI* 95% Confidence interval, *Ref* Reference group. Bold values, Statistical significance with *p < 0.05, **p < 0.01, ***p < 0.001

### Interaction effect of cancer worry across age on self-reported depressive symptoms (Model III)

**Fig. 1:**
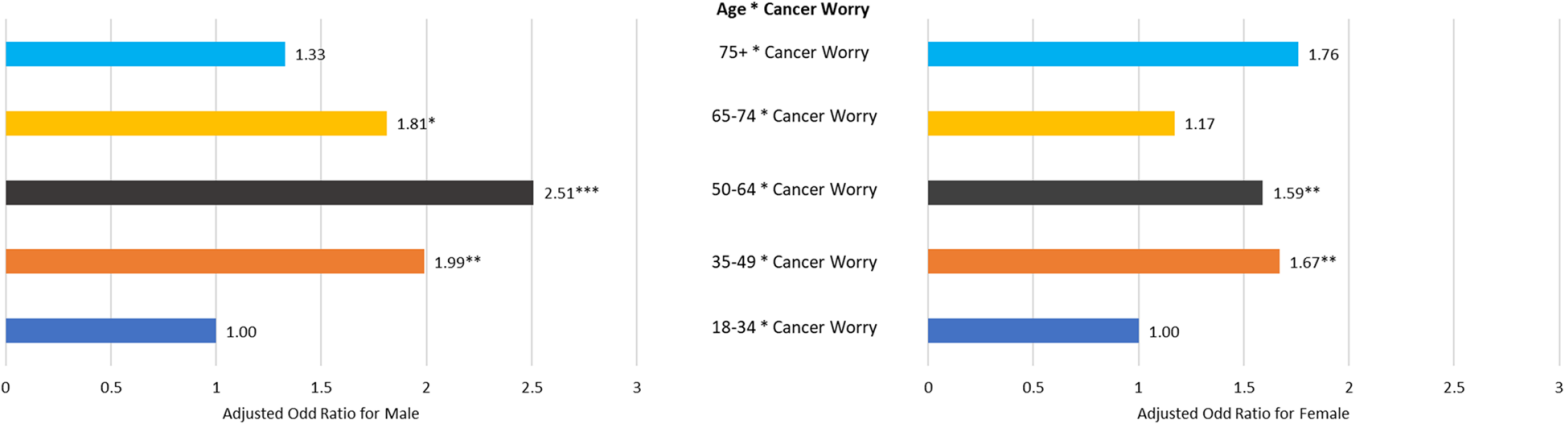
Model III: Self-reported depressive symptoms association with the interaction of cancer worry across age and sex: Adjusted for confounders. HINTS 5 Cycle 4, 2020 data. Statistical significance: *p < 0.05, **p < 0.01, ***p < 0.001

When examining the interaction between individual’s age and CW, we found that, all age groups for both males and females had an increased likelihood of experiencing depressive symptoms compared with those aged 18–34 years, (males: AOR = 1.33–2.51 vs. females: AOR = 1.17–1.76). Among males, those aged 50–64 years with CW exhibited the highest likelihood of SRDS (AOR = 2.51, 95% CI = 1.69–3.73), while among females, those aged 75 years and older showed the highest association (AOR = 1.76, 95% CI = 0.98–3.18), although this was not statistically significant. Overall, the interaction model shows that males who experience CW were more likely than females to experience major depressive symptoms across all age groups, except those aged 75 years and older (See Fig. 1**)**.

## Discussion

This study examined the prevalence, the statistical difference, and the likelihood of SRDS association with CW and its interaction across age groups, while adjusting for other confounding factors, within and between both males and females. Our findings revealed that females had a higher prevalence rate of SRDS than males (32.0% vs. 23.5%), which is consistent with previous studies conducted by Brett Silverstein (1999) and Rebecca L. Rohde (2018) [27, 28]. We also observed a statistically significant difference in SRDS between males and females. Furthermore, we identified a statistically significant difference in SRDS among male and female subgroups based on CW and some confounders (i.e., age, marital status, sexual orientation, annual household income, and smoking status; including level of education among males, and race/ethnicity among females). In addition, we found some levels of CW and confounders (age, homosexual/gay/lesbian, bisexual, annual household income, and smoking) were significantly associated with SRDS, which is consistent with previous studies [29–31]. Notably, this is the first study to examine the interaction effect of age and CW on SRDS among US adults, and we found a statistically significant association between CW and SRDS across ages 35–74 years in males and 35–64 years in females.

Worry about cancer was found to be significantly associated with SRDS, when examining the independent factors with males being at higher risk than females, although both genders were at increased risk of SRDS. This is consistent with a study conducted by Peter. et al. (2001) which found that women who perceived worries about breast cancer reported higher levels of anxiety and confusion [32], supporting our findings. However, the relationship between cancer-related worry and health behaviors is complex, given that CW may result in both negative and positive health consequences. For example, Nathan S. et al. (2008) found in their study that trait anxiety was not related to screening, but worry about getting prostate cancer was found to be associated with frequent screening [33]. Similarly, in a study on psychological side effects of breast cancer screening found that women with suspicious abnormal mammograms had significantly increased mammography-related anxiety and breast cancer worries, despite ruling out breast cancer through diagnostic work-ups, which interfered with their moods and functioning [34]. Another study found a significant association between worry about breast cancer and the intention to obtain mammogram screening among various subgroups of women who underutilize screening [7]. Moreover, patients who have passed a cancer diagnosis within a year have reported higher levels of anxiety and worry about seeing a new physician and what their examination test would show [35]. In contrast, a study using a multidimensional scaling found that cancer-related worry was separated from anxiety, depression, and posttraumatic disorder symptoms [36], which contradicts our findings. Despite these conflicting evidences, most studies, including ours have demonstrated that CW is associated with depressive symptoms, which can influence health behaviors and outcomes. Therefore, understanding how CW influences depressive symptoms and health behaviors is crucial, and adopting strategies to reduce CW may enhance positive outcomes while reducing mental health problems.

In addition, the interaction of CW with different age groups was associated with an increased likelihood of SRDS across all genders and age ranges. Specifically, we found that the likelihood of SRDS increased among all ages who reported CW, compared with those aged 18–34 years. For instance, among females with CW, those aged 75 years and older had the highest increased likelihood (i.e.,76%, but not statistically significant) of SRDS. But among males with CW, those aged 50–64 years had the highest (i.e., 151%) increased odds of experiencing SRDS. This is consistent with other studies that reported aging to be associated with depressive symptoms [37–39]. Interestingly, when we examined age as a risk factor separately, we found that the prevalence and likelihood of SRDS generally decreased with age in both males and females, contradicting findings from previous studies [37, 38]. This suggests that other factors may be influencing the increasing association between age and depressive symptoms, as shown in this study and supported by the fact that cancer has been reported to be more prevalent in older age groups [40, 41]. Nonetheless, the depression rate among young adults has been reported to be increasing over the past decades in a most recent study by Thapar et al. (2022) [42]. The authors further reported that, young individuals who have a family history of depression, social stressor exposures, and subgroups like sexual minority and having a chronic physical health problem were at high risk of depressive symptoms. This may further explain the increasing SRDS among young adults. Further, across all age groups, males had higher decreased likelihood for SRDS than females. However, when we considered the interaction of age with CW, males were found to have higher increased likelihood for SRDS than females across all age groups, except those aged 75 years and older. McQueen et al. (2008) reported that men had a greater comparative perceived risk for developing cancers, while women experienced more frequent worry [13]. This may explain the reason why males with CW had higher SRDS than females. The authors further reported that worry about cancer varies, and several associations were moderated by gender. This study found several variations in SRDS between males and females, and while females mostly had a higher prevalence of SRDS than males, males were at relatively higher risk of SRDS than females when factoring in CW. A similar finding was reported by a previous study on gender differences in depression among six European Countries [17].

A study on age and depression by John Mirowsky and Catherine E. Ross (1992) [37] noted that late-life depression often arises due to losses in marriage, employment, economic well-being, physical dysfunction, and low personal control in addition to personal and status losses. [37] Conversely, depression tends to decline in early adulthood as a result of life gains. [37] On the other hand, Blazer D. et al. (1991) [39] found a reversed association between age and depression after controlling for factors such as being female, having lower income, physical disability, cognitive impairment, and social support. That is, as a person ages, depressive symptoms are likely to be less severe if factors associated with both increased age and depressive symptoms are simultaneously considered and addressed. It is unclear why such contrasting findings about the influence of age on depressive symptoms exist between males and females. Subsequently, further study is needed to better understand these dynamics in SRDS. Notwithstanding, this study has shown that CW may play a pivotal role in influencing SRDS. With cancer being the second deadliest disease in the world, often resulting from risk factors such as people’s lifestyle, environmental factors, family history, aging, etc., it is no coincidence that CW was associated with SRDS across the age groups. Therefore, addressing this through some form of education and cancer screening practices may help reduce SRDS which is a growing mental health problem.

### Policy implication

Depressive symptoms can pose serious medical illnesses with mood, cognitive, and physical. They have been found to be associated with higher rates of impaired functioning, chronic diseases, and increased healthcare utilization [43]. However, treatment turns out to be low and often inadequate [44]. Our study provided several policy implications that may aid the treatment of SRDS and improve mental health disorders. The findings from this study have shown that CW was associated with higher depressive symptoms among both males and females. More importantly, this is the first study that assessed the likelihood of the interaction of age with CW influence on SRDS. It was found that the interaction of age with CW had a tremendous relative increased risk of SRDS. Several studies have found that depressive symptoms increase the risk of depression. A systematic review of 57 studies assessing the risk of increased mortality among patients with depressive symptoms found a positive association in 51% of cases [45]. Depression has been found to be associated with a 50% increase costs of chronic illness [43]. Both major depressive symptoms and depression are linked to increased morbidity and mortality due to adverse psychological and health effects [43]. CW may be inevitable owing to the stressful life events associated with cancer, such as financial distress, suppression, repression, dissociation, and stigma [11]. Our study highlighted the impact of aging and CW, gender differences, and their interactions on SRDS. Understanding these factors is essential to aid in the prevention and treatment of mental health disorders, reducing chronic diseases and mortality among affected populations. Streamlining cancer interventional policies that can reduce cancer anxiety may help reduce CW. For instance, speeding up cancer screening, diagnosis, and treatment programs can result in reducing the waiting times of participating individuals or patients from being overwhelmed [46], which may reduce CW, hence reducing the possibility of developing depressive symptoms. Additionally, providing an interventional program that limits cancer anxiety [47] through tailored education and providing easily accessible educational materials on cancer and its screening may help reduce CW leading to depression.

### Limitations

Beyond the strength and importance of this study, there were some limitations. With a cross-sectional survey data used for this study, we are limited in making strong, accurate, or definitive conclusions from our findings. There are always biases associated with cross-sectional study design, so we must be cautious in interpreting and implementing the findings, especially for policy intervention. Most often, we recommend further studies to support and validate findings. In addition, the outcome of interest (depressive symptoms) was self-reported, hence, it is subject to response biases from remembering/recalling. We are not sure whether participants in the survey were also medically diagnosed with depressive symptoms, rather than based on just their feelings, which can be subjective. This potential bias could influence the strength of evidence of our findings. Therefore, the application of findings in this study must be approached with caution. Additionally, our study focuses on cancer worries in general. However, there may be cancer-specific worries, as different cancers and stages may present different worries. This can be considered for future studies. Finally, the present study did not include other confounding variables of CW and SRDS such as adverse life events.

## Conclusion

This study has shown that differences exist in the magnitude and impact of risk factors’ influence on SRDS among males and females. Even when a risk factor had a similar impact on SRDS such as increased likelihood/association, the extent/magnitude of the impact differed by gender. These further increase knowledge and understanding about gender differences in SRDS, which is important for the prevention and treatment of mental health diseases. It was found that CW is associated with an increased risk of SRDS among both males and females, with males more likely at risk; especially as age interacts with CW, which resulted in a tremendous increase in the likelihood of depressive symptoms. The CW has the tendency of causing unpleasant emotions and feelings which can consequently influence people’s physical and psychological functioning, resulting in mental health or psychiatric disorders. Our findings provide an understanding of the high-risk group of SRDS among males and females, which may help facilitate and speed-up the prevention of mental health diseases or outcomes. It further highlights the complex interplay between age, gender, and CW in relation to SRDS, and contributes to the growing body of literature on gender differences in depression. In summary, our findings suggest that CW and age interact to influence the likelihood of SRDS, with varying patterns observed among different age groups and genders. Further research is warranted to better understand the underlying mechanisms driving these associations and to inform targeted interventions for individuals at risk of depressive symptoms.

## Data Availability

The data are publicly available on the National Cancer Institute (https://hints.cancer.gov). Our analyzed data is available from the corresponding authors upon reasonable request.

## Abbreviations

AOR: Adjusted Odds Ratio
CI: Confidence Interval
CW: Cancer Worry
HINTS: Health Information National Trends Survey
NCI: National Cancer Institute
PHQ: Patient Health Questionnaire
Ref: Reference Group
SRDS: Self-Reported Depressive Symptoms
